# The impact of a private sector living wage intervention on consumption and cardiovascular disease risk factors in a middle income country

**DOI:** 10.1186/s12889-018-5052-2

**Published:** 2018-01-25

**Authors:** David H. Rehkopf, Katharine Burmaster, John C. Landefeld, Sarah Adler-Milstein, Emily P. Flynn, Maria Cecilia Acevedo, Jessica C. Jones-Smith, Nancy Adler, Lia C. H. Fernald

**Affiliations:** 1 Division of Primary Care & Population Health, Stanford University, Stanford, CA USA; 2 Department of Internal Medicine, Legacy Health, Portland, OR USA; 3 Department of Medicine, University of California San Francisco, San Francisco, CA USA; 4 Worker Rights Consortium, Washington, DC USA; 5 Swedish Medical Center, Seattle, WA USA; 6 Inter-American Development Bank, Washington, DC USA; 7 Department of Health Services & Epidemiology, University of Washington School of Public Health, Seattle, WA USA; 8 Center for Health and Community, University of California San Francisco, San Francisco, CA USA; 9 Division of Community Health Sciences, School of Public Health, University of California, Berkeley, Berkeley, CA USA

**Keywords:** Diet, Blood pressure, Obesity, Occupational health, Socio-economic

## Abstract

**Background:**

A positive association of socioeconomic position and health is well established in high-income countries. In poorer nations, however, higher income individuals often have more cardiovascular risk factors (including obesity) than do those with less income. Our study goal was to estimate the effects of receiving a living wage (340% higher income) on short-term changes in consumption and cardiovascular risk factors among low-wage workers in a middle-income country.

**Methods:**

This cross-sectional study matched workers at an apparel factory (*n*=105) in the Dominican Republic with those at a similar factory (*n*=99) nearby, 15 months after the intervention factory introduced a substantially higher living wage. Statistical matching on non-time varying individual characteristics (childhood health, childhood living conditions, work experience, demographic factors) strengthened causal inference. Primary outcomes were blood pressure (systolic and diastolic), pulse rate, body mass index and waist circumference. Secondary outcomes were dietary consumption and spending on services, consumables and durable goods.

**Results:**

Receiving the living wage was associated with increased consumption of protein, dairy, soda and juice and sugars, but not with cardiovascular risk factors. Intervention factory workers spent more on grocery items and household durable goods.

**Conclusions:**

While having a higher income in a middle-income country might be expected to increase obesity and its associated health risks, the current study found no short-term negative associations. There may be possible longer-term negative health consequences of increases in consumption of soda, juice and sugars, however. It is important to consider complementary interventions to support healthy dietary intake in areas with increasing wages.

**Electronic supplementary material:**

The online version of this article (10.1186/s12889-018-5052-2) contains supplementary material, which is available to authorized users.

## Background

Given the existence of a socioeconomic gradient for diseases including cardiovascular disease [1], one might expect that raising the income of those in poverty would improve health. The exception, however, may be in low and middle-income countries (LMIC) where inverse associations of socioeconomic status and cardiovascular risk factors have been found. For example, countries below $2500 per capita gross domestic product (GDP) tended to have higher rates of obesity among the wealthy, with the relationship reversed in countries with greater than $2500 per capita GDP [2]. In addition, the evaluation of interventions in Mexico and Columbia providing income to families via conditional cash transfer (CCT) programs have found that increased cash provided to the households was associated with increased risk for obesity in adults [3–5].

While these findings suggest the potential for deleterious impacts of income on cardiovascular risk factors in LMIC, there are several reasons that this question requires further investigation. First, the small number of prior investigations of effects of increased income on obesity, blood pressure and nutritional consumption have been based on governmental sponsored CCT programs, and effects may differ by type (and amount) of income intervention. In high-income countries, minimum wage laws have been used to increase the wages of workers. A complementary approach to public sector minimum wage ordinances are private sector interventions [6]. This includes paying workers a living wage, defined as a minimum income standard – the wage necessary to procure basic goods and services, rather than an arbitrary minimum wage. Differences in effects on health related factors may reflect differences in the nature of the income intervention itself [7]. Secondly, CCT programs that have been evaluated involve a much smaller increase in income. For example the CCTs in Columbia and Mexico represented a 20-30% increase among the poor, while living wages can increase income by more than 10 times that amount. Thirdly, although more than a dozen CCT programs exist, examining impacts on risk factors for cardiovascular health is very uncommon [8]. Finally, CCTs by definition require specific behavior changes – i.e. the “conditions” associated with the cash award – and thus not just an income intervention.

Investigating the impacts of an increase in income is critical to the 6 billion people living in LMICs, where the majority of the world’s poor live. Within the population of people living in poverty, cardiovascular disease (CVD) has emerged as a threat to overall health; three-quarters of global deaths due to CVD are in LMICs [9, 10]. In an analysis of countries worldwide, death rates from chronic disease were 54% and 86% higher for men and women in LMICs than in high-income countries [11]. While deaths due to infectious disease are still of primary importance and result in substantial mortality, in particular early in life, the emergence of CVD is a threat that also must be addressed.

We examine the association of participating in a living wage intervention with obesity and cardiovascular risk factors. In two previously published papers, we have reported the positive effects of this living wage intervention on reducing depressive symptoms and improving self-reported health [12, 13]. Here we test whether these benefits extend to cardiovascular diseases or whether cardiovascular risk is increased. We examined BMI, waist circumference, systolic blood pressure, diastolic blood pressure and pulse. We also examined differences in consumption patterns in order to better understand the mechanisms through which changes in cardiovascular risk factors could occur.

## Methods

### Data

Our intervention sample was workers at an apparel factory that opened in April of 2010, and hired 130 individuals from a small city in the Dominican Republic. Prior to opening the factory, the owners of the factory decided to pay workers a living wage, which was not disclosed to workers applying for jobs at the factory. The initial monthly wage was set at RD$18153 which was 340% higher than the legal minimum wage in the free trade zone of RD$5400. A single factory for obtaining the ‘comparison’ study sample was chosen because it best fit predetermined criteria for a matched factory: it manufactured apparel, was of a similar size, was less than 50 miles from the intervention factory and was located within a city of a similar size and population distribution.

The data from this study come from an in-person survey and a physical exam administered in the workers’ homes in July and August of 2011. Of the 107 eligible workers at the intervention site at the introduction of the living wage, 105 completed surveys (2 refused to participate). Six of these stopped working at the factory during the 15 month period prior to data collection, bringing the intervention factory sample to 99 workers. Of 132 eligible workers at the comparison site, 105 completed surveys (18 were untraceable, 5 were no longer working, 3 were absent when surveys were conducted, and 1 declined to participate). Thus, the final sample consisted of 204 workers: 99 workers from the treatment site and 105 workers from the comparison site.

### Matching variables

In order to control for possible baseline differences between the workers in the intervention factory and the comparison factory, we matched on factors that could be associated with differences in the outcomes we examined:

#### Childhood health and deprivation

Participants were asked “In the first 15 years of your life, was your health: excellent, good, normal, bad or very bad.” “Excellent”, “good” and “normal” were collapsed together as good health. They were also asked whether there was a bathroom or latrine inside their childhood home and whether the home had electricity in the first 15 years of life; response options were “always,” “sometimes” or “never,” which we recoded into two categories of “always” and “sometimes” or “never”.

#### Demographics

We also matched on gender, years of work experience, and years of education. Of all matching variables only height was missing some observations: 7 individuals were missing height and 2 had biologically implausible values recorded (< 80cm). These 9 individuals were assigned the mean level of height by gender in our sample (158.5 cm for women, 169.4 cm for men) for matching purposes in order that these observations were not dropped from all analyses.

### Consumption and expenditure outcomes

#### Finances

Monthly household income and monthly savings were self-reported. Total debt was calculated by summing self-reported amounts of all of a participant’s current debts and loans. Information was also collected about whether participants had taken out any loans in the past year, the purpose(s) of the loan(s), the source(s) of the loan(s), and if the participant was behind on any payments.

For recurrent expenses, participants were asked to report how often they spent money on a particular category of expenses and how much they spent on average each time. For annual expenses, participants were asked to report how much money they spent on a particular category of expenses in the past 12 months.

#### Diet

Participants reported the number of times various types of food were consumed during the last seven days using a food frequency questionnaire [14]. For dietary consumption we removed implausible reports of consumption that were more than 5 standard deviations away from the mean (we removed 1 value for healthy carbohydrates, 1 value for vegetables, 2 values for soda and juice and 1 value for sugars).

### Cardiovascular risk factor outcomes

Participants were weighed using a digital scale with two weights taken to the tenth of a kilogram; height was measured without shoes using a stadiometer and measured twice to the nearest mm, using a standardized protocol [15]. Waist circumference was measured twice to the nearest mm with the tape measure snug but not compressing the skin. Blood pressure was measured using an Omron HEM-790 automated blood pressure monitor. Participants were seated, and had not smoked, had alcohol or been physically active in the 30 minutes prior to the measurement. Two blood pressure and pulse rate readings were taken 1 minute apart; the average of the two readings for each of these measures was used. Obesity was defined as BMI ≥30, overweight as BMI ≥25, high blood pressure as systolic≥120 mmHg or diastolic≥80 mmHg, tachycardia≥85 beat per minute, and high waist circumference as ≥85 cm for women and ≥90 cm for men [16].

### Statistical methods

We use a statistical matching approach for making inference from our data because there is unlikely to naturally be exact matching between individuals in the intervention and comparison factories with respect to factors that may be related to the outcomes we examine [17]. We matched on the following eight characteristics which occurred prior to the study exposure and were hypothesized based on prior knowledge from the literature to potentially be associated with the exposure or outcomes: age, gender, height, years of formal education, years of work experience, good childhood health, childhood in a home with a latrine and childhood in a home with electricity. We use an algorithmic matching method, ‘genetic matching’ to calculate the optimal match across these 8 criteria [18]. This method has similarities to the more commonly used propensity score and Mahalanobis distance approaches but has the advantage of nonparametric matching to optimizing the matching algorithm across covariates [18]. This model choice does not involve the outcomes, but only involves selecting the best model for how the eight potential confounding factors predict the treatment. We use the “matching” [19] and the “Rgenoud” package [20] in the R statistical environment for our analyses.

After matching, we estimated the group mean of each outcome of interest and calculated a t-test statistic to determine whether the difference would be expected by chance. In sensitivity analysis we found that the quality of matching using ‘genetic matching’ in our data was slightly better than using an alternative propensity score approach, and thus present the findings using ‘genetic match’ (data not shown). However, we also present in supplemental material our results using a propensity score approach implemented using the “MatchIt” package in R [21].

While our primary results are presented in the population as a whole, systematic analyses have shown that the relationship between anthropometric factors and income may differ by gender [22]. Thus we also fit gender stratified models of the relationship between treatment and cardiovascular risk factors.

## Results

### Comparison of the characteristics of the intervention and comparison factories

Table 1 shows the characteristics of the intervention and comparison factories before and after matching, and a statistical test of differences between the levels of each variable for the treatment factory and the comparison factory.

**Table 1:** Comparison of worker characteristics in the living wage factory and the comparison factory prior to and after statistical matching

	Before matching			After matching	
	Living wage factory	Comparison factory	*p*-value	Comparison factory	*p*-value
Age	35	34	0.025	36	0.073
Female	0.76	0.57	0.0031	0.75	0.83
Height	162	162	0.89	161	0.32
Formal education	3.7	4.6	0.95	3.1	0.050
Work experience	13	13	0.42	13	0.80
Good childhood health	0.61	0.68	0.27	0.72	0.10
Latrine in childhood	0.13	0.21	0.15	0.22	0.09
Electricity in childhood	0.70	0.54	0.017	0.72	0.75

Table notes: Age is in years, height is in centimeters, formal education is in years, work experience is in years. Female, poor childhood health, latrine in childhood and electricity in childhood are dichotomous measures – values in table are prevalence proportion

The Additional file 1: Figure S1 presents a comparison of the intervention and comparison factories before matching, showing generally similar characteristics with the exception of childhood electricity and gender. The matching resulted in a much closer balance by gender and electricity in childhood. Because the goal of the matching algorithm is to find particular individuals in the comparison factory that are the best overall match with each individual in the treatment factories, some characteristics appear on average to be less similar after matching.

### Impacts of intervention on economic outcomes

Table 2 shows the impact of the intervention on household earnings, savings and debt.

**Table 2:** Matched analysis of the effects of the living wage factory on household earnings, savings and debt.

	Treatment Effect	Standard error	T-statistic	*p*-value
Income and savings				
Monthly household income (RD$)	13454	2460	5.5	<0.001
Monthly savings (RD$)	2578	714	3.6	<0.001
Debt				
Total debt (RD$)	19737	4611	4.3	<0.001
Loan source is bank	66	0.23	2.8	0.0054
Overdue payments on debt	-38	0.11	3.4	<0.001

Table notes: Values for monthly household income, monthly savings and total debt are shown in Dominican pesos. Overdue payments on debt is for the past year and is among individuals who have debt (*n*=198). Loan source is bank is a yes/no variable and is among individuals reporting having a loan (*n*=104). Treatment effect is an absolute difference taken by subtracting the mean in the control factory from the mean of the treatment factory. For loan source is bank and overdue payment on debt, differences are in percentage points.

As expected, monthly household income was much higher for intervention factory workers. Intervention workers also had significantly higher levels of monthly savings. Although intervention workers also reported more debt than did comparisons, the nature of the debt was different: loans to intervention workers were 66 percentage points more likely to be from a bank and 38 percentage points less likely to be overdue on debt payments compared to workers in the comparison factory.

### Impacts of intervention on spending and consumption

Table 3 shows treatment effects for both spending and dietary consumption.

**Table 3:** Matched analysis of the effects of the living wage factory on consumption

	Treatment Effect	Standard error	T-statistic	*p*-value
Diet				
Healthy carbohydrates	-0.0030	0.20	0.015	0.99
Vegetables	0.20	0.19	1.1	0.29
Fruits	-0.11	0.20	0.52	0.60
Protein	0.67	0.20	3.3	<0.001
Dairy	0.83	0.20	4.2	<0.001
Soda and juice	0.46	0.16	2.8	0.0047
Sugars	0.27	0.11	2.5	0.013
Services spending				
School fees (RD$)	2177	1436	1.5	0.13
Consumable spending				
Grocery/supermarket (RD$)	59795	16204	3.7	<0.001
Prepared food (RD$)	-5826	6491	0.90	0.37
Other food (RD$)	10778	8563	1.3	0.21
School materials (RD$)	2125	68356	0.031	0.975
Transportation (RD$)	-6577	22300	0.29	0.768
Durable good spending				
Furniture/appliances (RD$)	9732	1615	6.0	<0.001
Car/motorcycle (RD$)	-4731	4707	1.0	0.31
Computer (RD$)	2265	848	2.7	0.0075
Property (RD$)	4687	4134	1.1	0.25
Home repair (RD$)	12528	4881	2.6	0.010

Table notes: Dietary measures are frequency of consumption (number of times per week) Z-scored so treatment effect is in terms of standard deviation. Services, consumable and durable good spending treatment effects are presented as a difference between the mean in the treatment factory and the mean of the control factory. RD$ is Dominican pesos. At the time of the study the exchange rate was 1 US dollar to 38 RD$; 1 Euro to 49 RD$; 1 British Pound to 57 RD$

In addition to greater savings, reported in Table 2, we found significantly greater spending on some types of expenditures.

Specifically, there was significantly more household spending at the grocery store, on furniture and home appliances, on computers and related technology and on home repair for households with individuals working at the living wage factory.

Reflecting greater spending on groceries, we found significantly greater reported consumption of several types of foods in the intervention group. The greatest difference was in consumption of dairy and protein, 0.83 and 0.67 standard deviations higher in the living wage factory. Smaller differences were found in consumption of soda and juices and sugars.

### Impacts of intervention on cardiovascular risk factors

Table 4 shows estimates of treatment effects on clinical cut-points and continuous measures of cardiovascular risk factors.

**Table 4:** Matched analyses of the effects of the living wage factory on worker cardiovascular risk factors

	Treatment effect	Standard error	T-statistic	*p*-value
Clinical cut-points				
High blood pressure	-2.0	6.9	0.29	0.78
High pulse rate	-7.6	9.7	0.78	0.43
Obese	8.1	8.4	0.96	0.33
Overweight	11	10	1.1	0.27
Large waist circumference	-1.0	8.9	0.11	0.91
Continuous measures				
Systolic blood pressure (mm Hg)	-2.9	2.9	1.0	0.32
Diastolic blood pressure (mm Hg)	-2.1	2.5	0.85	0.40
Pulse rate (beats per minute)	-1.2	2.6	0.47	0.63
BMI (kg/m^2^)	1.4	1.1	1.3	0.20
Waist circumference (cm)	-0.072	2.2	0.032	0.97

Table notes: Treatment effect is the difference between the mean in the control factory subtracted from the mean of the treatment factory. Frequencies for clinical cut-points were: high blood pressure (*n*=34); high pulse rate (*n*=50); obese (*n*=34); overweight (*n*=95); large waist circumference (*n*=110). Obesity was defined as BMI ≥ 30, overweight as BMI ≥ 25, high blood pressure as systolic ≥ 120 mmHg or diastolic ≥ 80 mmHg, tachycardia as ≥85 beats per minute, and high waist circumference as ≥ 85 cm for women and ≥ 90 cm for men

There were no differences found between the treatment and comparison factories for any of the risk factors we examined.

### Supplemental analyses

While we did not have statistical power to test for effect measure modification by gender, we did test for associations within gender strata (data not shown). A notable association was found for pulse where there were lower levels in the treatment factory for women (-8.8, *p* = 0.0071) but not for men (3.3, *p* = 0.45). We found similar differences based on the clinical cut-point for tachycardia (Odds ratio=0.74, p=0.0062 for women; Odds ratio=1.34, *p* = 0.035 for men). We did not find substantial or statistically significant differences between the treatment and comparison factories within gender groups for any of the other cardiovascular risk factors.

We also examined all of our findings using an alternative statistical matching approach [23, 24] in order to test whether any of our findings were dependent on the particular matching algorithm (see Additional file 1: Tables S1-S3). Findings were similar.

## Discussion

We examined the impacts of a workplace intervention involving low-wage apparel workers in the Dominican Republic, who were paid a living wage of RD$18153 per month as compared to the prevailing minimum wage of RD$5400 per month, and the world bank poverty level equivalent of RD$1500 per month. We found no negative impacts on any of the measures of cardio-metabolic risk for the intervention versus the comparison workers. In terms of spending, we found that households in the intervention group were more likely to spend money on groceries (especially protein, dairy and, to a lesser extent, soda and juices), furniture and home appliances, computers and related technology, and on home repair.

The absence of differences in BMI and waist circumference between the groups is notable, and in contrast with prior literature on CCT programs. Given the greater expenditure on groceries and consumption of sodas, juices and sugars by the intervention workers and the positive association in LMICs between socioeconomic status and obesity, it is encouraging that the living wage workers did not show higher rates of obesity. That said, the effects of increased consumption of soda, juices and sugars may not emerge until later, beyond our 15-month follow-up period. The finding that there were even stronger differences in consumption of dairy products and protein suggests that the additional funds may have been used in a more balanced manner that did not promote excessive weight gain. It will be important, however, to monitor changes over time to determine if longer-term detrimental dietary patterns emerge and create health risks. The health of the workers may benefit from both local and national programs focused on improving dietary consumption in the context of economic development in order to optimize the overall benefits of living wage interventions in middle-income countries [25, 26].

Taken together with previously reported findings of a reduction of depressive symptoms and a significant increase in self-rated health among those employed in the intervention factory [25, 26], these findings are encouraging. The positive impacts on savings, protein and household consumption are similar to those found in prior studies of CCT programs [27, 28]. There are also, however, differences found as compared to other studies that demonstrate the context dependent nature of income based interventions [7], including differences in the effects on debt [27], and differences in the effects on fruit and vegetable consumption [28].

Analyzing the impact of a private sector living wage involves analytic challenges since workers are not randomly assigned to the living wage condition. However, several factors work against selection bias. First, workers did not know at the time they applied for employment that they would be paid a living wage; the hiring pool was thus very similar to that of the comparison factory. Secondly, we chose a comparison factory that was similar on key characteristics to the intervention factory. We also analytically matched on eight individual characteristics that occurred prior to the intervention and that could be related to cardiovascular disease risk.

Our study, however, is limited by the fact that this intervention took place in only one factory and the number of workers in this factory limited our statistical power. Low statistical power could account for the lack of detected differences in cardio-metabolic risk. It is important to emphasize that the evaluation of impacts is for 15 months, and it is unknown whether obesity will increase over longer periods of time. However, prior work in Mexico did find impacts on obesity after just 23 months, and the fact that in this study no adverse impacts were found among women who were not yet overweight at baseline suggests that the lower prevalence of overweight individuals in our population at the time of intervention could be important for explaining the difference in findings [4]. Finally, this intervention took place in one LMIC, the Dominican Republic (gross national income per capita is approximately $6,000). LMICs are extremely diverse socially and economically. How and why living wage interventions diminish or exacerbate cardiovascular disease must be understood in each different context [29].

The findings of this study are encouraging in terms of overall positive effects of efforts to pay a living wage to workers. Workers receiving a higher wage saved more and invested more in goods (e.g. computers, furniture and appliances and home repair), which could raise their standard of living in a more sustained way. They also spent more in grocery markets and had greater intake of protein and dairy products. Greater consumption of protein and dairy can have positive impacts on health, although future work should examine the specific sources of protein consumption given the potential for negative health and environmental consequences of animal based protein [30]. In addition, we found higher levels of spending on sodas, juices and sugars in the living wage factory. The association with cardiovascular risk, whether measured as a continuous variable or in terms of clinically-meaningful cut-offs, were not statistically significant. However, we did observe a greater consumption of sodas/juices and sugar.

It is important to emphasize that while we do not observe differences in cardiovascular risk between the treatment and control factories, these differences may emerge over a longer period of time. Considering our results on higher levels of soda/juice and sugar consumption in the living wage factory, our study suggests the need for policies to promote healthier diets in conjunction with increases wages. While countries at all income levels need to address the increasing incidence of obesity and diabetes in their populations, this may be of the greatest importance in countries undergoing increases in wages, or in locations where living wages are implemented [31, 32]. Several types of interventions may be of use, including approaches that focus on availability of types of food as well as food prices and taxation [33–35]. Given the fact that our living wage intervention was employer based, an additional actionable approach could involve workplace-based changes to address specific aspects of dietary intake [36, 37].

## Conclusions

Conditional cash transfer programs in low and middle income countries have shown benefits for some health outcomes, but several studies have shown that there are increases in obesity. No previous research has examined the effects of a living wage intervention in a low or middle income country on dietary consumption and cardio-metabolic risk factors, including obesity. We found that following the implementation of the living wage policy at the factory, there was increased consumption of protein, dairy, soda and juice and sugars, but not with cardiovascular risk factors. In consideration with other beneficial impacts of the living wage, it suggests that there is an overall benefit on health and development, but with potential harm for long term cardio-metabolic health from increased soda, juice and sugar consumption.

## Additional file

Additional file 1: Sensitivity analyses. Table S1, S2 and S3 and Figure S1. (DOCX 109 kb)

## Abbreviations

BMI: Body mass index
CCT: Conditional cash transfer
CM: Centimeters
CVD: Cardiovascular disease
GDP: Gross domestic product
LMIC: Low and middle-income countries
Mm: Millimeters
MmHg: Millimeters of mercury
RD$: Dominican pesos
