# Supplementary material for: The impact of a private sector living wage intervention on consumption and cardiovascular disease risk factors in a middle income country
Source: BMC Public Health. 2018 Jan 25;18:179. doi: 10.1186/s12889-018-5052-2 (PMC5785889; doi:10.1186/s12889-018-5052-2)

**Additional File 1**

Supplemental Table 1. Matched analysis (using MatchIt and Zelig) of the effects of the living wage factory on household earnings, savings and debt.

|  | **Treatment**  **Effect** | **Standard**  **deviation** | **Lower 95% CI** | **Upper 95% CI** |
| --- | --- | --- | --- | --- |
| Income and savings |  |  |  |  |
| Monthly household income (RD$) | 12657 | 2024 | 8887 | 16678 |
| Monthly savings (RD$) | 1823 | 509 | 829 | 2796 |
| Debt |  |  |  |  |
| Total debt (RD$) | 14780 | 3343 | 8406 | 21171 |
| Loan source is bank | 70 | 0.40 | -0.11 | 1.46 |
| Overdue payments on debt | -32 | 0.076 | -0.47 | -0.18 |

Values for monthly household income, monthly savings and total debt are shown in Dominican pesos. Overdue payments on debt is for the past year and is among individuals who have debt (n=198). Loan source is bank is a yes/no variable and is among individuals reporting having a loan (n=104). Treatment effect is an absolute difference taken by subtracting the mean in the control factory from the mean of the treatment factory. For loan source is bank and overdue payment on debt, differences are in percentage points.

Supplemental Table 2. Matched analysis (using MatchIt and Zelig) of the effects of the living wage factory on consumption.

|  | **Treatment**  **Effect** | **Standard**  **deviation** | **Lower 95% CI** | **Upper 95% CI** |
| --- | --- | --- | --- | --- |
| Diet |  |  |  |  |
| Healthy carbohydrates | -0.176 | 0.14 | -0.47 | 0.072 |
| Vegetables | 0.25 | 0.12 | 0.006 | 0.48 |
| Fruits | -0.056 | 0.17 | -0.38 | 0.26 |
| Protein | 0.52 | 0.15 | 0.22 | 0.82 |
| Dairy | 0.68 | 0.15 | 0.40 | 1.00 |
| Soda and juice | 0.36 | 0.10 | 0.17 | 0.56 |
| Sugars | 0.047 | 0.10 | -0.16 | 0.25 |
| Services spending | |  |  |  |
| School fees (RD$) | 2887 | 1097 | 822 | 4953 |
| Consumable spending | | | | |
| Grocery/supermarket (RD$) | 42774 | 15135 | 13827 | 73089 |
| Prepared food (RD$) | 5032 | 4265 | -3467 | 13276 |
| Other food (RD$) | 10412 | 9391 | -7656 | 28557 |
| School materials (RD$) | 7757 | 45316 | -80658 | 89364 |
| Transportation (RD$) | 18847 | 14497 | -8754 | 46730 |
| Durable good spending | | | | |
| Furniture/appliances (RD$) | 8727 | 1239 | 6340 | 11247 |
| Car/motorcycle (RD$) | 1597 | 2848 | -3978 | 7465 |
| Computer (RD$) | 1954 | 615 | 792 | 3131 |
| Property (RD$) | 4751 | 3079 | -1218 | 10928 |
| Home repair (RD$) | 12229 | 3958 | 3865 | 19681 |

Dietary measures are frequency of consumption (number of times per week) Z-scored so treatment effect is in terms of standard deviation. Services, consumable and durable good spending treatment effects are presented as a difference between the mean in the treatment factory and the mean of the control factory. RD$ is Dominican pesos. At the time of the study the exchange rate was 1 US dollar to 38 RD$; 1 Euro to 49 RD$; 1 British Pound to 57 RD$.

Supplemental Table 3. Matched analysis (using MatchIt and Zelig) of the effects of the living wage factory on worker cardiovascular risk factors.

|  | **Treatment**  **Effect** | **Standard**  **Deviation** | **Lower 95% CI** | **Upper 95% CI** |
| --- | --- | --- | --- | --- |
| Clinical cut-points |  |  |  |  |
| High blood pressure | -8.5 | 5.4 | -19 | 2.3 |
| High pulse rate | -3.2 | 6.4 | -16 | 9.0 |
| Obese | 6.8 | 5.3 | -3.7 | 17 |
| Overweight | 3.7 | 7.5 | -11 | 18 |
| Large waist circumference | -4.1 | 7.1 | -18 | 11 |
| Continuous measures |  |  |  |  |
| Systolic blood pressure (mm Hg) | -2.7 | 2.2 | -6.9 | 1.5 |
| Diastolic blood pressure (mm Hg) | -0.54 | 1.7 | -3.8 | 2.9 |
| Pulse rate (mm Hg) | -1.3 | 1.7 | -4.6 | 2.1 |
| BMI (kg / m^2^) | 0.82 | 0.75 | -0.73 | 2.2 |
| Waist circumference (cm) | -1.8 | 2.1 | -5.8 | 2.3 |

Treatment effect is the difference between the mean in the control factory subtracted from the mean of the treatment factory. Frequencies for clinical cut-points were: high blood pressure (n=34); high pulse rate (n=50); obese (n=34); overweight (n=95); large waist circumference (n=110). Obesity was defined as BMI≥30, overweight as BMI≥25, high blood pressure as systolic≥120 or diastolic≥80, tachycardia as≥85, and high waist circumference as ≥85 cm for women and ≥90cm for men.

Supplemental Figure. Comparison of distribution of pre-intervention characteristics between intervention factory (blue) and comparison factory (red).


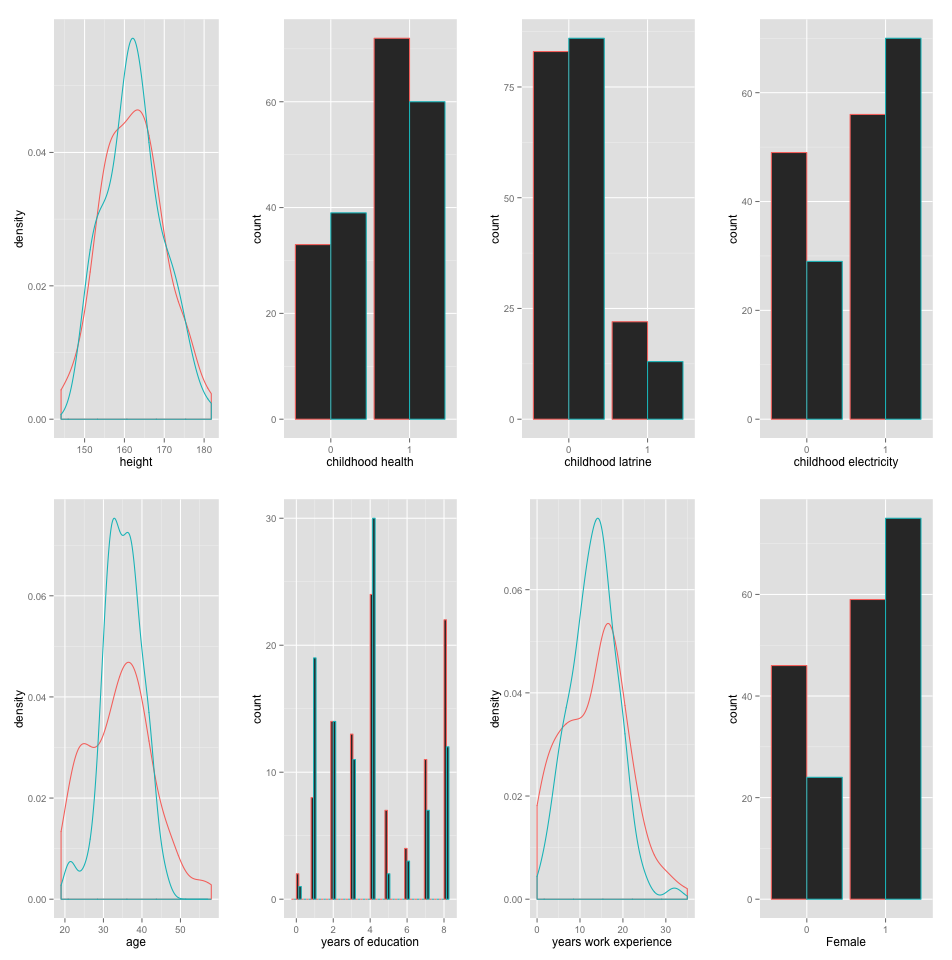

Supplement: Additional file 1: — Sensitivity analyses. Table S1, S2 and S3 and Figure S1. (DOCX 109 kb) [file 12889_2018_5052_MOESM1_ESM.docx]
